# Emerging SARS-CoV-2 Mutational Variants of Concern Should Not Vary in Susceptibility to Microbicidal Actives

**DOI:** 10.3390/life12070987

**Published:** 2022-06-30

**Authors:** M. Khalid Ijaz, Raymond W. Nims, Julie McKinney

**Affiliations:** 1 Global Research and Development for Lysol and Dettol, Reckitt Benckiser LLC, One Phillips Drive, Montvale, NJ 07645, USA; juliemckinney@reckitt.com; 2 RMC Pharmaceutical Solutions, Longmont, CO 80501, USA; rnims@rmcpharma.com

**Keywords:** coronavirus, COVID-19, decontamination of surfaces and hands, Beta variant, Delta variant, formulated microbicidal active, hierarchy of microbicidal susceptibility to pathogens, SARS-CoV-2, viral inactivation, virucidal efficacy

## Abstract

The severe acute respiratory syndrome coronavirus 2 (SARS-CoV-2) is evolving, with emergence of mutational variants due to the error-prone replication process of RNA viruses, in general. More recently, the Delta and Omicron variants (including sub-variants BA.1–5) predominate globally, and a Delta–Omicron recombinant termed Deltacron has emerged. The emergence of variants of concern (VOC) demonstrating immune evasion and potentially greater transmissibility and virulence naturally raises concern in both the infection control communities and the public at large, as to the continued suitability of interventions intended to mitigate the risk of viral dissemination and acquisition of the associated disease COVID-19. We evaluated the virucidal efficacy of targeted surface hygiene products (an ethanol/quaternary ammonium compound (QAC)-containing disinfectant spray, a QAC disinfectant wipe, a lactic acid disinfectant wipe, and a citric acid disinfectant wipe) through both theoretical arguments and empirical testing using international standard methodologies (ASTM E1053-20 hard surface test and EN14476:2013+A2:2019 suspension test) in the presence of soil loads simulating patients’ bodily secretions/excretions containing shed virus. The results demonstrate, as expected, complete infectious viral inactivation (≥3.0 to ≥4.7 log_10_ reduction in infectious virus titer after as little as 15 s contact time at room temperature) by these surface hygiene agents of the original SARS-CoV-2 isolate and its Beta and Delta VOC. Through appropriate practices of targeted surface hygiene, it is expected that irrespective of the SARS-CoV-2 VOC encountered as the current pandemic unfolds (and, for that matter, any emerging and/or re-emerging enveloped virus), the chain of infection from virus-contaminated fomites to the hand and mucous membranes of a susceptible person may be disrupted.

## 1. Introduction

The severe acute respiratory syndrome coronavirus 2 (SARS-CoV-2) is evolving. The emergence of mutational variants is not unexpected, and these mutations are attributed to the error-prone replication process of RNA viruses in general. The SARS-CoV-2/COVID-19 pandemic has caused (as of 31 May 2022) ~530 million confirmed cases globally and ~6.3 million deaths [1]. Accordingly, the disease has been, and remains, the focus of global scientists and public health authorities, including the US Centers for Disease Prevention and Control (CDC) [2,3] and the World Health Organization (WHO) [4]. Some of the SARS-CoV-2 variants are considered Variants of Concern (VOC) [5] because they are more transmissible and/or virulent [6] compared with the SARS-CoV-2 originally reported in Wuhan, China. More recently, SARS-CoV-2 variants predominating globally have included the Omicron variant (and sub-variants BA.1–5) [5], and the Delta–Omicron recombinant termed Deltacron [7], which has recently been reported to exhibit greater immune-evasion (reduced immune-mediated neutralization) compared to the Omicron sublineages and Delta variant [8].

The emergence of VOC during the ongoing SARS-CoV-2 pandemic naturally raises concern in both the infection control communities, and the public at large, as to the continued suitability of interventions intended to mitigate the risk of viral dissemination and acquisition of the associated disease COVID-19. For instance, questions may arise about the efficacy of vaccines against SARS-CoV-2 and the efficacy of targeted hygiene approaches for interrupting the cycle of infection of SARS-CoV-2 (i.e., from contaminated surfaces or hands to susceptible mucous membranes). Will those interventions effective for SARS-CoV-2 also be effective for the emerging VOC? Will targeted hygiene approaches effective for SARS-CoV-2 be effective against other pre- and post-pandemic emerging respiratory viruses [9] (e.g., new strains of influenza or respiratory syncytial virus)?

Evidence is beginning to accumulate demonstrating that individuals vaccinated against SARS-CoV-2 are acquiring infections with the Delta and Omicron variants [10,11]. The immune evasion of the Delta and Omicron variants in individuals vaccinated and boosted with the current SARS-CoV-2 vaccines [12] is alarming. Additionally, mutations in variants may not be limited to the spike protein against which the commonly used SARS-CoV-2 vaccines were raised, as these have been observed in the nucleocapsid protein, suggesting that variant-specific vaccines may be required in the future as the virus continues to evolve [13]. These concerns over vaccination efficacy only serve to increase the importance of non-pharmaceutical interventions, including mask wearing, social distancing, contact tracing, and hand and surface hygiene for infection prevention and control (IPAC).

**Figure 1:**
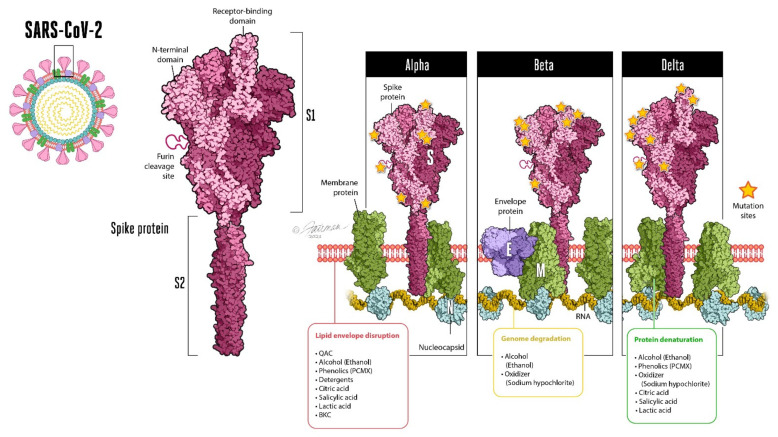
Schematic representation of SARS-CoV-2, showing mutation sites for the Alpha, Beta, and Delta variants [5] and sites of action for different classes of microbicidal actives (modified, from [15,18]). Note: The Omicron variant (not shown) contains more than 45 mutations in the spike protein [17]. Inactivation of enveloped viruses by formulated microbicidal actives and detergents is believed [15,18] to result from (1) disruption of the viral phospholipid bilayer glycoproteinaceous envelope, (2) denaturing of viral proteins, and (3) degradation of the viral genome.

Fortunately, the virucidal efficacy of surface and hand hygiene agents should not be impacted by the mutations being observed in emerging SARS-CoV-2 variants, since this susceptibility is determined by the commonalities in mechanisms of action of the formulated microbicidal actives against enveloped viruses in general [14,15,16]. The virucidal activity of microbicides involves multiple distinct mechanisms (Figure 1), including the disruption of the viral envelope, denaturation of the viral envelope-containing spike proteins (the receptor-binding domain required for virus–host cell interactions), and/or genomic degradation. Note that these mechanisms should apply to enveloped viruses in general [14,15,16], and to SARS-CoV-2 and its emerging variants, which currently differ primarily with respect to mutations within different regions of the spike and/or nucleocapsid proteins [13]. The Omicron variant contains more than 45 mutations in the spike protein [17], the receptor-binding domain of the virus which leads to virus–host interactions initiating host–cell infection.

## 2. Methods

### 2.1. Challenge Viruses, Host Cell Lines, and Reagents

Virucidal efficacy testing against SARS-CoV-2 (Wuhan isolate and Beta and Delta variants) was performed for microbicidal active-containing surface hygiene products per standardized methods [19,20]. Details on the challenge viruses and their sources and the detector (host) cell line used for propagation of viral stocks and for cell-based infectivity (titration) assays are displayed in Table 1. This table also indicates the culture media used in these assays.

### 2.2. Standardized Efficacy Testing Methodologies

Virucidal efficacy evaluations of formulated microbicidal actives against coronaviruses experimentally deposited on a non-porous glass surface were conducted per ASTM E1053-20 [19]. The active ingredient concentrations, contact times, and exposure temperatures evaluated, and the organic soil load are indicated in Table 2. For each test run, an aliquot of 0.4 mL of the challenge viral fluid (virus plus soil load) was added onto a pre-sterilized 10-cm glass Petri dish and spread over the entire surface of the dish. The virus was allowed to dry at ambient temperature. Then, 2.0 mL of test spray was added onto the dried viral film by direct spray, such that the dried virus film was completely covered by the test microbicide. In the case of wipes, the glass carriers contaminated with virus were subjected to three passes of wiping with the wipe. The dishes were held for 15 s at 20 ± 1 °C, then 2.0 mL of neutralizer (for citric acid: MEM + 10% newborn calf serum + 3% HEPES + 1% NaHCO_3_ + 0.5% polysorbate 80 + 0.025 N NaOH; for ethanol/quaternary ammonium compounds (QAC): MEM + 10% newborn calf serum + 0.5% polysorbate 80 + 0.5% lecithin + 2% HEPES + 0.025 N HCl; for QAC: MEM + 10% newborn calf serum + 0.5% polysorbate 80 + 0.5% lecithin) were added onto the dishes and the viral inoculum/test microbicide/neutralizer mixture was scraped off the dish using a cell scraper. Various cytotoxicity and neutralization controls were used according to the method [19]. The quenched samples were serially ten-fold diluted in dilution medium (MEM + 2% newborn calf serum) and inoculated onto host cells to assay for infectious virus using 50% tissue culture infectious dose (TCID_50_) assay.

Virucidal efficacy evaluations of formulated microbicidal actives suspended in liquid matrices were conducted per EN 14476:2013 + A2:2019 [20]. The challenge matrix was cell culture medium (Table 1) containing various organic loads (Table 2). The active ingredient concentrations tested, contact times, and exposure temperatures evaluated, and the organic soil loads employed are indicated in Table 2. For each run, one part of the challenge virus was added to eight parts of the test product solution expressed from the wipes, which was pre-equilibrated to ambient temperature, in the presence of one part of soil load (5% bovine serum or 3% BSA and 3% erythrocytes), and immediately mixed thoroughly via a vortex mixer. The test mixtures were maintained at the contact temperature for 5 min, then immediately collected and quenched in neutralizer (ice-cold MEM + 10% newborn calf serum + 2% HEPES + 0.01 N NaOH) to reduce cytotoxicity to the host cells. Various cytotoxicity and neutralization controls were used according to the method [20]. Neutralized test samples were serially ten-fold diluted in dilution medium (MEM + 2% newborn calf serum) and inoculated onto host cells to assay for infectious virus using the TCID_50_ assay.

## 3. Results and Discussion

Testing of surface hygiene products, including a spray disinfectant and three disinfecting wipes containing different microbicidal actives, demonstrated equivalent virucidal efficacy (≥3 log_10_ reduction) against SARS-CoV-2 and its Beta and Delta variants (Table 2). The disinfectant spray and citric acid or QAC pre-impregnated wipes were tested using the hard-surface methodology (ASTM E1053-20 [19]). In each case, complete inactivation of the challenge virus was obtained for SARS-CoV-2 or its variants dried on a prototypic (glass) hard surface. In the suspension test (EN 14476:2013 + A2:2019 [20]) used for the lactic acid pre-impregnated wipes, complete inactivation of the challenge virus was obtained, even in the case where the soil load consisted of bovine serum albumin and erythrocytes (an especially stringent inactivation matrix more closely simulating human bodily fluids). These data demonstrate that the SARS-CoV-2 Wuhan isolate and the Beta and Delta variants are similarly susceptible to these surface hygiene agents. Although this result might have been expected (see below), it is always of value to empirically confirm virucidal efficacy expectations.

A variety of formulated microbicidal actives used for targeted surface and hand hygiene previously have been evaluated using international testing standards [16]. These included alcohol-, quaternary ammonium compound-, hydrochloric acid-, organic acid-, p-chloro-m-xylenol-, and sodium hypochlorite-based microbicidal formulations. Only minor differences in virucidal efficacy against different alpha- and beta-coronaviruses, including SARS-CoV-2, were observed in those studies [16]. A recent article by Meister et al. [21] also demonstrated that personal care products (a hand soap and various concentrations of ethanol), when tested in suspension inactivation studies, resulted in similar virucidal efficacies for the original SARS-CoV-2 isolate (B.1.1.70) and the Delta (B.1.1.7) and Beta (B.1.351) variants. According to the hierarchy of susceptibility of pathogens to microbicides [14,15,16], all enveloped viruses, including SARS-CoV-2, newly emerging VOC including the Delta and Omicron variants, other coronaviruses, as well as enveloped viruses from other virus families, should be susceptible to these microbicides (reviewed in [22]). In fact, this concept led the U.S. Environmental Protection Agency (EPA) to activate an Emerging Viral Pathogen (EVP) Guidance for SARS-CoV-2 on 29 January 2020 [23], and more recently for monkeypox in May 2022 [24], intending to facilitate IPAC in cases where empirical virucidal efficacy data for an emerging virus may be lacking. The EPA extended its EVP policy indefinitely to include SARS-CoV-2 and its variants on 19 November 2021 [24]. 

The outcome of the present study, which was conducted according to standardized methodologies [19,20] under especially stringent testing conditions (different challenging soil loads and virus dried on prototypic hard surfaces), confirm and extend our earlier observations related to the virucidal efficacy of these microbicides for coronaviruses in general, and SARS-CoV-2 and its variants in particular. Taken together, the above considerations provide confidence in targeted surface hygiene agents, empirically demonstrated to possess virucidal efficacy against SARS-CoV-2, for IPAC of newly emerging SARS-CoV-2 variants, such as the Delta and Omicron variants, or Deltacron, as well as for other pre- and post-pandemic emerging enveloped respiratory viruses [16,22]. These observations should be useful to the global IPAC community as it deals with the newly emerging Omicron variant and sub-variants BA.1–5, which are spreading rapidly globally [25]. The Omicron variant contains more than 45 mutations in the spike protein (receptor binding domain) of the virus, which leads to virus–host interactions initiating host–cell infection. Many of these changes have been found in other VOC (e.g., in the Delta variant) and are linked to increased transmissibility/infectivity and immune evasion. The impact of the mutations on virulence for the Omicron variant [25,26,27,28,29,30] remains to be determined. Where and how these SARS-CoV-2 variants are emerging is not totally clear. Preliminary data suggest that SARS-CoV-2 persists for months in immunocompromised patients (e.g., those infected with human immunodeficiency virus, or recipients of organ transplants). Under such circumstances, the virus may acquire mutations over time, leading in some cases to more highly transmissible VOC which are able to evade immune surveillance/neutralization and to propagate in human cells [26].

Arguments have been made that the primary transmission route for SARS-CoV-2 and other respiratory viruses involves respiratory droplets [31,32], with the indirect route involving virus-contaminated surfaces being relegated to a less important role. These arguments deserve additional scrutiny [33,34]. Both the World Health Organization [35] and the U.S. Centers for Disease Prevention and Control [36] have acknowledged that the prevention of transmission of respiratory pathogens involves interruption of contact, droplet, and airborne transmission. Contaminated fomites (high-touch environmental surfaces or HITES) represent intermediate surfaces or objects which may contribute to the direct contact route. That is, these intermediate fomites lie between the infected and the susceptible persons [37]. Interruption of the chain of infection, therefore, should be possible through effective disinfection of contaminated HITES, through disruption of the surface–hand touch network [37,38,39]. 

Through appropriate practice of targeted hygiene, it is expected that irrespective of the SARS-CoV-2 VOC or emerging enveloped respiratory viruses encountered post-pandemic (resurgence in influenza and RSV has been reported [22,40]), the chain of infection from virus-contaminated fomites to the hand and mucous membranes of a susceptible person may be interrupted. This conclusion becomes of importance as new variants of SARS-CoV-2 emerge in the ongoing pandemic, or with re-emergence of post-pandemic respiratory viruses [41].

## Figures and Tables

**Table 1:** Challenge viruses, detector (host) cell lines, and reagents used *.

Species	Isolate	Strain	Source	Host Cell	Source	Description	Culture Medium
SARS-CoV-2	Wuhan	Isolate USA-WA1/2020	CDC, through BEI Resources NR-52281	Vero E6	ATCC CRL-1586	African green monkey kidney	MEM+ 5% FBS
SARS-CoV-2	Beta VOC	hCoV-19/South Africa/KRISP-EC-K005321/2020 Lineage B.1.351	CDC, through BEI Resources NR-54008				
SARS-CoV-2	Delta VOC	hCoV-19/USA/PHC658/2021 Lineage B.1.617.2	CDC, through BEI Resources NR-55611				
SARS-CoV-2	Delta VOC	hCoV-19/England/204820464/2020 (UK/VUI/3/2020) Lineage B 1.1.7	CDC, through BEI Resources NR-54000				

* Abbreviations used: ATCC, American Type Culture Collection; CDC, U.S. Centers for Disease Control and Prevention; CoV, coronavirus; FBS, fetal bovine serum; MEM, minimal essential medium.

**Table 2:** Comparison of virucidal efficacy of surface hygiene products against SARS-CoV-2 and its VOC at room temperature ^a^.

Microbicidal Product Type (Active)	Contact Time	Temperature (°C)	Relative Humidity (%)	Organic Load	Log_10_ Reduction in Infectious Virus Titer ^h^		
					SARS-CoV-2	Delta VOC	Beta VOC ^g^
Disinfectant spray (Ethanol (50%)/ QAC (0.086%)) ^b,c^	15 s	20 ± 1	33–36	5% Bovine serum	≥4.6, ≥4.7, ≥4.5	≥4.0, ≥4.0 ^e^	≥4.0, ≥4.0
Wipe (Lactic acid (3.2%)) ^d^	5 min	20 ± 1	Not recorded	BSA (3%), erythrocytes (3%) ^j^	Not tested	≥4.75 ^e^	≥4.50
Wipe (Lactic acid (3.2%)) ^d^	5 min	18	55	5% Bovine serum	≥4.6	Not tested	Not tested
Wipe (Citric acid (2.5%)) ^b^	15 s	20 ± 1	36–40	5% Bovine serum	≥3.0, ≥3.0, ≥3.0	≥3.75, ≥3.75 ^e^	≥4.0, ≥4.0
Wipe (QAC (0.2%)) ^b,i^	15 s	20 ± 1	36–47	5% Bovine serum	≥3.5, ≥3.5, ≥3.5	≥4.0, ≥4.0 ^f^	≥3.75, ≥3.75

^a^ Abbreviations used: BSA, bovine serum albumin; QAC, quaternary ammonium compound; SARS-CoV-2, severe acute respiratory syndrome coronavirus-2; VOC, variant of concern. ^b^ Tested per ASTM E1053-20 Standard [19] on a glass surface. ^c^ QAC: Alkyl (50% C14, 40% C12, 10% C16) dimethyl benzyl ammonium saccharinate. ^d^ Tested per EN14476:2013 + A2:2019 Standard [20] in suspension. ^e^ Lineage B.1.1.7 (Delta variant). ^f^ Lineage B.1.167.2 (Delta variant). ^g^ Lineage B.1.315 (Beta variant). ^h^ Where multiple values are shown, these represent testing of independent product lots. Where inactivation was found to be complete to the limit of detection, the values are indicated as “≥”. Data for SARS-CoV-2 are from reference [16] and are displayed for the purpose of comparison. ^i^ QAC: Alkyl (50% C14, 40% C12, 10% C16) dimethyl benzyl ammonium chloride. ^j^ The presence of erythrocytes and BSA represents a greater challenge for inactivation.

## Data Availability

Additional information, other than that shown in Table 1 and Table 2, may be obtained from the corresponding author.
